# Associations Between *CYP17A1* and *SERPINA6/A1* Polymorphisms, and Cardiometabolic Risk Factors in Black South Africans

**DOI:** 10.3389/fgene.2021.687335

**Published:** 2021-08-13

**Authors:** Siphiwe N. Dlamini, Ananyo Choudhury, Michèle Ramsay, Lisa K. Micklesfield, Shane A. Norris, Nigel J. Crowther, Andrew A. Crawford, Brian R. Walker, Zané Lombard, Julia H. Goedecke

**Affiliations:** ^1^South African Medical Research Council/Wits Developmental Pathways for Health Research Unit, Department of Paediatrics, Faculty of Health Sciences, University of the Witwatersrand, Johannesburg, South Africa; ^2^Non-communicable Diseases Research Unit, South African Medical Research Council, Cape Town, South Africa; ^3^Faculty of Health Sciences, Sydney Brenner Institute for Molecular Bioscience, University of the Witwatersrand, Johannesburg, South Africa; ^4^Department of Chemical Pathology, National Health Laboratory Service, Faculty of Health Sciences, University of the Witwatersrand, Johannesburg, South Africa; ^5^Population Health Sciences, Bristol Medical School, University of Bristol, Bristol, United Kingdom; ^6^BHF Centre for Cardiovascular Science, University of Edinburgh, Edinburgh, United Kingdom; ^7^Translational and Clinical Research Institute, Newcastle University, Newcastle upon Tyne, United Kingdom; ^8^Division of Human Genetics, National Health Laboratory Service, Faculty of Health Sciences, School of Pathology, University of the Witwatersrand, Johannesburg, South Africa

**Keywords:** *SERPINA6*, *SERPINA1*, *CYP17A1*, metabolic syndrome, blood pressure, lipids, insulin, cortisol

## Abstract

Research in European and Asian populations has reported associations between single nucleotide polymorphisms (SNPs) in *CYP17A1* and *SERPINA6/A1* and circulating glucocorticoid concentrations, and some key cardiometabolic risk factors. This study aimed to investigate these associations in black South African adults, who are disproportionally affected by the metabolic syndrome and its related cardiometabolic risk factors. The dataset included black South African adults (*n* = 4,431; 56.7% women) from the AWI-Gen study, genotyped on the H3A genotyping array and imputed using the African reference panel at the Sanger imputation service. From the imputed data, 31 *CYP17A1* SNPs and 550 *SERPINA6/A1* SNPs were extracted. The metabolic syndrome and its components were defined using the 2009 harmonized guidelines. Serum glucocorticoid concentrations were measured in a subset of 304 men and 573 women, using a liquid chromatography-mass spectrometry method. Genetic associations were detected using PLINK. Bonferroni correction was used to control for multiple testing. A SNP at *SERPINA6/A1*, rs17090691 (effect allele G), was associated with higher diastolic blood pressure (BP) in all adults combined (*p* = 9.47 × 10^−6^). Sex-stratified analyses demonstrated an association between rs1051052 (effect allele G), another *SERPINA6/A1* SNP, and higher high-density lipoprotein (HDL) cholesterol concentrations in women (*p* = 1.23 × 10^−5^). No association was observed between these variants and glucocorticoids or between any of the *CYP17A1* SNPs and metabolic outcomes after adjusting for multiple testing. Furthermore, there were no associations between any of the SNPs tested and the metabolic syndrome. This study reports novel genetic associations between two SNPs at *SERPINA6/A1* and key cardiometabolic risk factors in black South Africans. Future replication and functional studies in larger populations are required to confirm the role of the identified SNPs in the metabolic syndrome and assess if these associations are mediated by circulating glucocorticoids.

## Introduction

Key cardiometabolic risk factors, including elevated waist circumference, blood pressure, and fasting blood glucose concentrations, and the presence of dyslipidemia, cluster to form the metabolic syndrome (Alberti et al., 2009). However, the prevalence of the metabolic syndrome generally differs by both sex and ethnicity, with urban-dwelling black South African women exhibiting a higher prevalence compared to their male counterparts, and women of other ethnic groups in similar urban settings (Goedecke et al., 2017; Gradidge and Crowther, 2017; Gerdts and Regitz-Zagrosek, 2019). The underlying mechanisms that explain these sex- and ethnic-based differences are unclear. Identifying biomarkers and common genetic variants that are associated with the metabolic syndrome and its related cardiometabolic risk factors, has the potential to improve the biological understanding of this condition and consequently the risk for common cardiometabolic diseases such as type 2 diabetes and cardiovascular disease.

Population-based cross-sectional studies have suggested that circulating cortisol, the primary glucocorticoid in humans, is associated with a higher risk of presenting with the metabolic syndrome and its related cardiometabolic risk factors (Walker et al., 2000; Reynolds et al., 2003; Ward et al., 2003). Likewise, single nucleotide polymorphisms (SNPs) within genes involved in glucocorticoid biology have been implicated in key components of the metabolic syndrome (Diver et al., 2016). SNPs in *CYP17A1* are reported to be associated with elevated blood pressure in Asians and Europeans (Levy et al., 2009; Kelly et al., 2013; Franceschini et al., 2016), and with adiposity in East Asians (Hotta et al., 2012). As the *CYP17A1* gene encodes an enzyme that converts precursors for corticosterone to precursors for cortisol (Yadav et al., 2017), the associations between *CYP17A1* SNPs and elevated blood pressure and adiposity are thought to be mediated by glucocorticoids (Diver et al., 2016). Considering that cardiometabolic risk factors that form the metabolic syndrome are interrelated (Alberti et al., 2009), the *CYP17A1* SNPs may also be associated with elevated fasting blood glucose and the presence of dyslipidemia, but this hypothesis is yet to be tested.

Further, in a genome-wide association study (GWAS) in Europeans, inter-individual variation in circulating cortisol concentrations was associated with SNPs at a locus that spans the *SERPINA6* and *SERPINA1* genes (Bolton et al., 2014). *SERPINA6* encodes corticosteroid binding globulin (CBG), the primary glucocorticoid-binding protein in circulation, while *SERPINA1* encodes alpha-1 antitrypsin, which is involved in the regulation of the binding and release of glucocorticoids from CBG (Henley and Lightman, 2011; Crawford et al., 2021). However, associations between SNPs at *SERPINA6/A1* and the metabolic syndrome and related cardiometabolic risk factors remain to be investigated.

The above-mentioned genetic studies were limited to non-Africans and only a few investigated sex-specific associations (Hotta et al., 2012; Bolton et al., 2014; Crawford et al., 2019). Genetic association studies in Africans have the potential to provide more effective localization of the disease-causing gene variants as a result of generally lower linkage disequilibrium in African genomes, compared to studies in populations of European ancestry (Gurdasani et al., 2015).

Hence, the primary aim of this study was to investigate the associations between *CYP171A* and *SERPINA6/A1* SNPs and the metabolic syndrome and its related cardiometabolic risk factors, in black South African men and women. The study also aimed to investigate the hypothesis that the observed genetic associations are mediated by circulating glucocorticoid concentrations.

## Materials and Methods

### Study Population

This study included participants from the Africa Wits-INDEPTH partnership for Genomic Studies (AWI-Gen) project. Briefly, AWI-Gen is a population-based study comprising over 12,000 adult men and women of African ancestry from six research centers in the west, east, and southern parts of Africa (Ramsay et al., 2016). This study was restricted to the AWI-Gen research centers that are based in South Africa: the Dikgale Health and Demographic Surveillance System (HDSS) in the Limpopo Province, MRC/Wits Agincourt HDSS in the Mpumalanga Province near the Mozambican border, and MRC/Wits Developmental Pathways for Health Research Unit (DPHRU) in Soweto (Johannesburg). Most of the female participants from the Soweto DPHRU research center were the caregivers of the Birth to Twenty Plus cohort, which is an ongoing longitudinal study previously described (Richter et al., 2007). Serum samples for glucocorticoid determination were only available from a subset of 877 participants (304 men and 573 women) from the Soweto site. Overall, the present study included 5,268 black South Africans (2,226 men and 3,042 women).

The AWI-Gen data collection was approved by the University of Witwatersrand Human Research Ethics Committee (Medical; Certificate numbers: M121029 and M170880). Collection of data and serum from the DPHRU sub-samples used for glucocorticoid determination were also approved by the same committee (Certificate numbers: M090620 and M160604). Likewise, the committee approved all secondary analyses described in the present study (Certificate number: M160225).

### Participant Testing Procedures

Collection of the AWI-Gen phenotype and related data was described in detail previously (Ali et al., 2018), and only variables relevant to this study are briefly described below.

#### Anthropometry, Blood Pressure, and Lifestyle Factors

Weight was measured to the nearest 0.1 kg using a digital scale (Kendon Medical, South Africa), and height was measured to the nearest millimeter using a Harpenden digital stadiometer (Holtain, Wales). Weight and height were subsequently used to calculate body mass index (BMI = weight in kg/height in m^2^) and obesity was classified as BMI ≥ 30 kg/m^2^. Waist circumference was measured to the nearest millimeter using a stretch-resistant soft measuring tape (SECA, Hamburg, Germany), at the level of the narrowest part of the torso, halfway between the lowest rib and the iliac crest.

Blood pressure was measured on the right arm using a digital blood pressure reader (Omron M6, Kyoto, Japan) and appropriately sized cuffs. Each participant rested for at least 5 min prior to the blood pressure measurement in a seated position. The blood pressure was measured in triplicate at 2-min intervals, but only the average of the second and third readings were recorded and used in the analyses.

In terms of lifestyle factors, smoking and alcohol status were determined by asking the participants if they smoked any form of tobacco product (e.g., cigarettes, cigars, or pipes), or consumed any form of alcoholic beverage (e.g., beer, wine, spirits, fermented cider, or traditional beer). The participants were subsequently classified as current smokers/non-smokers and drinkers/non-drinkers. The participants were asked to bring all their medications (Meds) to the interview sessions for confirmation and recording of chronic Meds used.

#### Blood Sampling and Biochemical Analyses

Participants fasted overnight for 10–12 h prior to collection of fasting blood samples. A standard venepuncture technique was used to collect the blood samples for determination of serum biomarkers (fasting glucose, insulin, and lipids), and to obtain buffy coat for DNA extraction. Fasting glucose and lipid concentrations were measured using the Randox Plus chemistry analyzer (Crumlin, Northern Ireland) by colorimetric assays. The intra-assay coefficient of variation (CV) for glucose was 2.3%, while the intra-assay CV for lipids were all less than 1.5%. The concentration of low-density lipoprotein (LDL) cholesterol was subsequently estimated using the Friedewald Equation (Friedewald et al., 1972). Fasting insulin concentrations were determined by an enzyme-linked chemiluminescent immunometric assay on the Immulite 1000 immunoassay system (Siemens, Hamburg, Germany) and the intra-assay CV was less than 2.0%. Homeostatic Model Assessment of Insulin Resistance (HOMA-IR) was subsequently computed from the fasting glucose and insulin concentrations, using the HOMA2-IR calculator version 2.2.3.^1^

Serum corticosterone and cortisol concentrations were determined on the subset of 877 participants from the Soweto DPHRU cohort. Selection of these participants was primarily based on availability of serum samples for glucocorticoid determination. The time of fasting was recorded and ranged between 7:30 am and 11:59 am. A liquid-liquid extraction technique (Agilent Technologies, California, United States) was used to extract the glucocorticoids. The glucocorticoid concentrations were subsequently analyzed by ultra-high-pressure liquid chromatography-mass spectrometry (UPLC-MS; Waters Corp, Milford, United States). The inter and intra-assay CVs were 7.3 and 2.9% for corticosterone, and 13.6 and 9.6% for cortisol, respectively.

### Definition of the Metabolic Syndrome

Presence of the metabolic syndrome was based on the 2009 harmonized criteria (Alberti et al., 2009). Briefly, the following cut off points were used to define components of the metabolic syndrome: (i) elevated waist circumference (≥94 cm in men and ≥80 cm in women); (ii) elevated triglycerides (≥1.7 mmol/L); (iii) low high-density lipoprotein (HDL) cholesterol (<1.0 mmol/L in men and <1.3 mmol/L in women); (iv) elevated blood pressure (≥130 mmHg for systolic and/or ≥85 mmHg for diastolic and/or using blood pressure medication); and (v) elevated glucose (≥5.6 mmol/L and/or using diabetes medication). Participants with three or more of the above components were classified as metabolic syndrome cases.

### Genotyping, Imputation, and Data Quality Control

DNA was extracted from buffy coats using a modified salting-out method (Miller et al., 1988) or the QIAsymphony SP (QIAGEN GmbH, QIAGEN Strasse 1, 40724 Hilden, Germany). The Human Heredity and Health in Africa (H3Africa) genotyping array (Illumina Inc., California, United States) was used to perform genome-wide genotyping of over 2.3 million SNPs in all AWI-Gen participants (Illumina FastTrack service). The H3Africa genotyping array was specifically designed to account for the larger genetic diversity and smaller haplotype blocks observed in the genomes of populations of African ancestry. Pre-imputation quality control (QC) steps included removal of participants and SNPs with high data missingness (>5%), SNPs with low minor allele frequency (MAF; <1%) and those not in Hardy-Weinberg Equilibrium (HWE; *p* < 1 × 10^−4^). Mitochondrial, non-autosomal, and ambiguous SNPs that failed to match the *GRCh37* reference alleles and strands were also removed.

The Sanger Imputation Server (McCarthy et al., 2016) was used to perform imputation on the cleaned dataset, which comprised of 1,729,661 SNPs and 10,903 participants from all AWI-Gen research centers. The African Genome Resources was used as the reference panel, EAGLE2 (Loh et al., 2016) for pre-phasing, and the default positional Burrows-Wheeler transform algorithm for the imputation (Rubinacci et al., 2020). Post-imputation QC steps included the removal of poorly imputed SNPs (info score < 0.6), SNPs with low MAF (<1%), and SNPs failing the HWE (*p* < 1 × 10^−4^). The final cleaned and imputed AWI-Gen dataset had 13.98 million SNPs.

From the imputed dataset, SNPs in the *CYP17A1* gene (all introns and exons) and in the 2 kb region upstream of the transcription start site of *CYP17A1* were included (*GRCh37* coordinates, 10:104590288–104599495), as SNPs in this region were previously associated with glucocorticoid concentrations and the metabolic syndrome in Europeans (Diver et al., 2016; MacKenzie et al., 2019). Secondly, all SNPs in the region from the start of transcription for the *SERPINA6* gene to the end of transcription for the *SERPINA1* gene including the region in between the two genes were also selected (*GRCh37* coordinates, 14:94769460–94857029). The intergenic region between *SERPINA6* and *SERPINA1* was included as it is known to harbor SNPs associated with circulating cortisol concentrations (Bolton et al., 2014). After removing the non-South African samples from the dataset, related participants were also removed (PI_HAT > 0.1875; *n* = 758 participants removed). Due to previously reported associations between glucocorticoid metabolism and critical illness (Boonen et al., 2013), participants previously diagnosed with cancer were also removed from the dataset (*n* = 79 participants removed). The final selected dataset used in the present analyses included 581 SNPs (31 from the *CYP17A1* and 550 from the *SERPINA6/A1* loci) in 4,431 black South Africans (1,918 men and 2,513 women).

### Statistical Methods

The Shapiro-Wilk test and histogram plots were used to assess the distribution of continuous variables in STATA v.13.1 software (Stata Corp. LLC, Texas, United States). Non-normally distributed outcome variables were first mathematically transformed (using either log or square root transformations) to obtain normality prior to inclusion in the linear regression models. The Mann-Whitney U and Chi-square tests were used to test statistical differences between men and women in the continuous and categorical variables, respectively.

All genetic association tests were performed using PLINK v.1.9 software (Purcell et al., 2007). Associations between the selected SNPs and the metabolic syndrome and its related cardiometabolic risk factors were tested using logistic and linear regression models, respectively. Similarly, linear regression models were also used to examine the associations between all SNPs and serum glucocorticoid concentrations in the subset of Soweto participants. Age, sex, smoking, alcohol consumption, and BMI (except where BMI, waist circumference, or the metabolic syndrome were the outcome variables) were included as potential confounders in all regression models. Due to the diurnal nature of glucocorticoids, blood sampling time was also included as an additional covariate in models, where serum glucocorticoid concentrations were the outcome variables. To minimize confounding due to prescribed medication, participants taking prescribed diabetes, blood pressure, or lipid medications (see Table 1 for numbers) were excluded from linear regression analyses involving glucose, insulin, blood pressure, or lipids, respectively, as outcomes.

**Table 1:** Characteristics of the study sample of black South Africans and a comparison between sexes.

Study sample	Main genetic association study sample				
	*N*	All	Men	Women	*p*
Age (years)	4,431	52 (46–58)	52 (46–58)	52 (46–58)	0.185
BMI (kg/m^2^)	4,147	27.2 (22.2–32.7)	23.2 (20.1–27.4)	30.5 (25.8–35.5)	<0.001
Waist circumference (cm)	4,148	95.8 (89.4–101.5)	88.0 (78.0–89.8)	98.0 (92.3–103.2)	<0.001
Systolic blood pressure (mmHg)	3,496	128.5 (116.0–144.0)	129.0 (116.5–144.0)	128.0 (115.5–144.0)	0.424
Diastolic blood pressure (mmHg)	3,499	83.0 (74.5–91.5)	84.0 (75.0–92.5)	82.5 (74.0–91.5)	0.027
Fasting glucose (mmol/L)	3,406	4.9 (4.6–5.4)	4.8 (4.4–5.3)	5.0 (4.6–5.5)	<0.001
Fasting insulin (uIU/ml)	3,417	7.2 (4.3–13.1)	7.0 (4.3–13.3)	7.4 (4.3–12.9)	0.327
HOMA-IR	2,985	1.6 (0.9–3.1)	1.6 (0.9–3.2)	1.7 (0.9–3.0)	0.768
Total cholesterol (mmol/L)	4,345	4.1 (3.5–4.9)	4.0 (3.3–4.7)	4.3 (3.6–5.0)	<0.001
LDL cholesterol (mmol/L)	4,309	2.5 (1.9–3.1)	2.3 (1.8–2.9)	2.6 (2.0–3.2)	<0.001
HDL cholesterol (mmol/L)	4,347	1.2 (1.0–1.4)	1.1 (0.9–1.4)	1.2 (1.0–1.4)	0.111
Triglycerides (mmol/L)	4,344	0.9 (0.7–1.3)	0.9 (0.6–1.3)	0.9 (0.7–1.3)	0.003
Blood pressure med [n/N (%)]	3,450	991/3,450 (28.7)	329/1,350 (24.3)	662/2,100 (31.5)	0.869
Diabetes med [n/N (%)]	3,450	366/3,450 (10.6)	134/1,362 (9.8)	232/2,088 (11.1)	0.259
Cholesterol med [n/N (%)]	2030	8/2,030 (0.4)	2/1,337 (0.1)	6/693 (0.9)	0.211
Smoking [n/N (%)]	3,108	597/3,108 (19.2)	538/1,078 (49.9)	59/2,030 (2.9)	<0.001
Alcohol [n/N (%)]	2,502	1,292/2,502 (51.6)	954/1,168 (81.7)	338/1,334 (25.3)	<0.001
^*^Corticosterone (nmol/L)	650	6.0 (3.5–12.2)	5.0 (3.4–8.8)	6.4 (3.6–14.6)	0.003
^*^Cortisol (nmol/L)	673	183.3 (96.6–322.6)	143.0 (74.1–295.7)	208.3 (84.2–343.8)	0.003
^*^Corticosterone/cortisol	548	0.05 (0.02–0.09)	0.04 (0.02–0.07)	0.05 (0.02–0.09)	0.050

Continuous data presented as median (IQR, interquartile range) and categorical data presented as number of prevalent/total number of observations (n/N) and percentage (%). Wilcoxon Rank Sum and Chi-square test were used to statistically compare the continuous and categorical variables, respectively, between the sex groups. *p*, value of *p* for the statistical difference between men and women; BMI, body mass index; HOMA-IR, homeostatic model assessment of insulin resistance; LDL, low density lipoprotein; HDL, high density lipoprotein; and Med, medication. All medication and lifestyle factors (smoking and alcohol) were self-reported during interview sessions.

* Measured in the Soweto sub-sample.

Bonferroni correction was used to control for multiple testing in all genetic association tests. The number of independent SNPs were determined with the “indep-pairwise” method in PLINK using the following thresholds: window size = 50 kilobase pairs, variance inflation factor = 5, and *r*^2^ = 0.5. The number of non-correlated outcomes (rho < 0.8) were determined using a Spearman’s correlation test. Hence, the Bonferroni adjusted *p*-value threshold was determined as *p* = 0.05/219 (number of independent SNPs)/10 (number of non-correlated outcomes) = 2.28 × 10^−5^. Sex interactions were also tested, and the sex interaction *p*-values were recorded as “Sex Int.” All models showing evidence of sex interaction (Sex Int < 0.05) were subsequently stratified by sex.

Overall, the outcomes investigated in this study included the metabolic syndrome and its individual components (waist circumference, systolic and diastolic blood pressure, fasting glucose, triglycerides, and HDL), and other related cardiometabolic risk factors (BMI, LDL, and total cholesterol, insulin, HOMA-IR), and circulating glucocorticoids (corticosterone, cortisol, and the corticosterone/cortisol ratio). Only models that showed evidence of association after adjusting for multiple testing (*p* < 2.28 × 10^−5^) and models that showed evidence of sex interactions (Sex Int < 0.05) are presented in the results.

## Results

### Study Sample Characteristics

**Figure 1:**
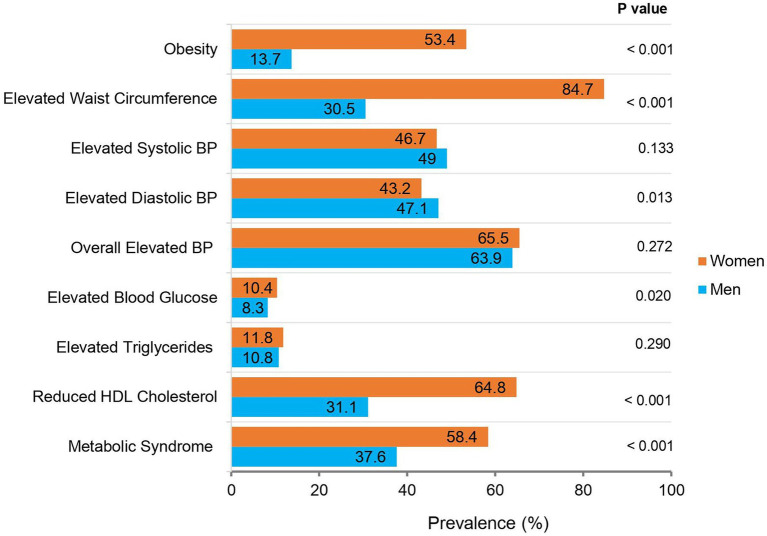
The prevalence of obesity, the metabolic syndrome and its components in black South African men and women (*n* = 4,431). *p*: Value of *p* for the statistical difference between men and women, using a chi-square test. BP, blood pressure. Obesity: Body mass index (BMI) ≥ 30 kg/m^2^; Elevated Waist Circumference: ≥94 cm in men and ≥80 cm in women; Elevated Systolic BP: ≥130 mmHg; Elevated Diastolic BP: ≥85 mmHg; Overall Elevated BP: Elevated Systolic BP and/or Diastolic BP and/or using BP medication; Elevated Blood Glucose: Elevated fasting glucose (≥5.6 mmol/L) and/or using diabetes medication; Elevated Triglycerides: Elevated fasting triglycerides (≥1.7 mmol/L); Reduced high-density lipoprotein (HDL) Cholesterol: Reduced fasting HDL-cholesterol (<1.0 mmol/L in men, <1.3 mmol/L in women); Metabolic Syndrome: 2009 Harmonized Criteria (Alberti et al., 2009).

The characteristics of the overall AWI-Gen black South African participants (main study sample) are presented and compared by sex in Table 1. The characteristics of the Soweto sub-sample on whom circulating glucocorticoids were measured, are compared to the main study sample in the Supplementary Table S1. Age, waist circumference, and alcohol consumption were not different; however, BMI and prevalence of smoking was higher in the glucocorticoid sub-sample compared to the main study sample. A comparison of the prevalence of obesity and the categorized individual components of the metabolic syndrome in the main study sample is presented in Figure 1.

There was no significant age difference between men and women in the main study sample. However, women had higher BMI and waist circumference compared to men (*p* < 0.001 for both). Accordingly, a greater proportion of women were obese and had elevated waist circumference compared to men (*p* < 0.001 for both). In contrast, men had higher diastolic blood pressure compared to women (*p* = 0.027), and this was accompanied by a greater proportion of men with elevated diastolic blood pressure compared to women (*p* = 0.013). There were no differences in systolic blood pressure, the use of blood pressure medication and the prevalence of overall elevated blood pressure (which considered both systolic and diastolic blood pressures and medication) between the sexes.

Women had higher fasting glucose (*p* < 0.001) and a greater proportion of the women had elevated glucose (*p* < 0.020) compared to men. In contrast, there were no differences in fasting insulin, HOMA2-IR, or the use of diabetes medication, between men and women. For circulating lipids, women had higher total and LDL cholesterol (*p* < 0.001 for both), and triglycerides (*p* = 0.003), and a greater proportion of women had low serum HDL cholesterol (*p* < 0.001), than men. There were no sex differences in elevated serum triglycerides or the use of lipid-lowering medication.

Corresponding to the above observations, a greater proportion of women were classified as having the metabolic syndrome compared to men (58.4 vs. 37.6%; *p* < 0.001). Conversely, a greater proportion of men smoked and consumed alcohol (*p* < 0.001 for both). In the sub-sample, women had higher corticosterone and cortisol concentrations compared to men (*p* = 0.003 for both). There was also a tendency for higher corticosterone/cortisol ratio in women compared to men (*p* = 0.050).

### Genetic Associations in Black South African Adults

**Figure 2:**
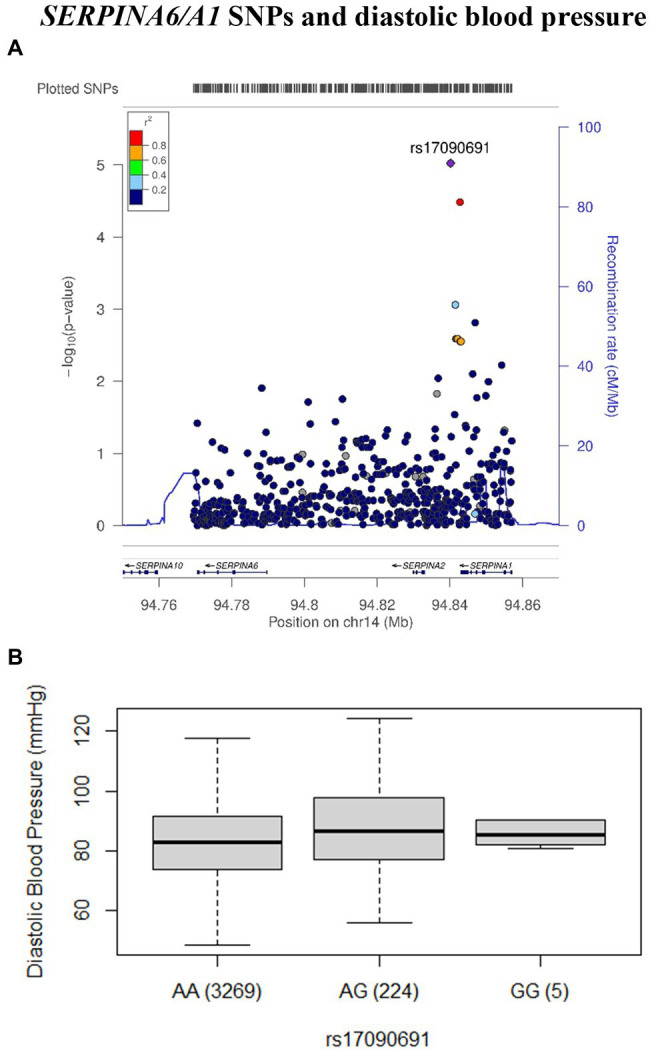
The association between rs17090691 and diastolic blood pressure in black South African men and women. The LocusZoom **(A)** plot association data was plotted using the AFR LD backgrounds from the 1,000 Genomes Project phase 1 dataset. The rs17090691 single nucleotide polymorphism (SNP) is represented by a purple diamond. Each SNP is colored according to the *r*^2^ value against rs17090691. SNPs with missing LD information are colored in gray. The box and whisker plot **(B)** shows medians and interquartile ranges (IQRs) of diastolic blood pressure across the rs17090691 genotype groups. The number of observations in each group is shown in brackets.

Although, none of the SNPs tested were associated with odds of presenting with the metabolic syndrome (Supplementary Table S2), rs17090691-G in the *SERPINA6/A1* region, showed an association with higher diastolic blood pressure in the sample comprising both sexes (Table 2). Figure 2A shows the LocusZoom summarizing association signals and LD in the surrounding region. The box and whisker plot that compares diastolic blood pressure across the rs17090691 genotype groups is shown in Figure 2B.

**Table 2:** Associations of *CYP17A1* and *SERPINA6/A1* SNPs in black South African men and women.

Phenotype	Locus	SNP	Minor (Effect) allele	Linear regression model adjusted for confounders		
				Beta (95% CI)	*p*	Sex Int
Fasting insulin	*CYP17A1*	rs10883783	A	−0.073 (−0.141, −0.005)	0.035	0.011
		rs743575	G	−0.072 (−0.140, −0.005)	0.036	0.009
		rs4919687	A	−0.072 (−0.139, −0.004)	0.037	0.010
		rs10883784	T	−0.067 (−0.134, 0.001)	0.053	0.006
		rs10786714	C	−0.067 (−0.135, 0.000)	0.051	0.005
HOMA-IR	*CYP17A1*	rs10883783	A	−0.085 (−0.157, −0.013)	0.021	0.033
		rs743575	G	−0.085 (−0.157, −0.014)	0.020	0.029
		rs4919687	A	−0.085 (−0.157, −0.013)	0.020	0.030
		rs10883784	T	−0.080 (−0.152, −0.008)	0.029	0.021
		rs10786714	C	−0.081 (−0.153, −0.009)	0.028	0.020
Fasting cortisol	*CYP17A1*	rs115978957	G	0.132 (0.032, 0.232)	0.010	0.047
		rs116032963	A	0.132 (0.032, 0.232)	0.010	0.047
Diastolic BP	*SERPINA6/A1*	rs17090691	G	0.065 (0.036, 0.094)	9.5 × 10^−6^	0.813
HDL cholesterol	*SERPINA6/A1*	rs60643124	G	−0.021 (−0.036, −0.006)	0.008	0.047
		rs12101216	T	−0.018 (−0.032, −0.003)	0.016	0.026
		rs74074941	T	−0.016 (−0.032, −0.001)	0.040	0.013
		rs74074947	T	−0.016 (−0.032, −0.001)	0.040	0.010
		rs58460454	T	−0.017 (−0.032, −0.001)	0.033	0.013
		rs1051052	G	0.016 (0.004, 0.028)	0.008	0.006

Linear regression models adjusted for age, sex, smoking, alcohol, and BMI (except where waist circumference was the outcome); Blood sampling time was included as an additional covariate where cortisol was the outcome. SNP, single nucleotide polymorphism; M, major allele; m, minor allele; Beta, unstandardized beta coefficient for the linear regression model; 95% CI, 95% confident intervals; HOMA-IR, homeostatic model assessment of insulin resistance; HDL, high-density lipoprotein; BP, blood pressure; *p*, value of *p* for the unstandardized beta coefficient; Sex Int, value of *p* for sex interaction.

**Figure 3:**
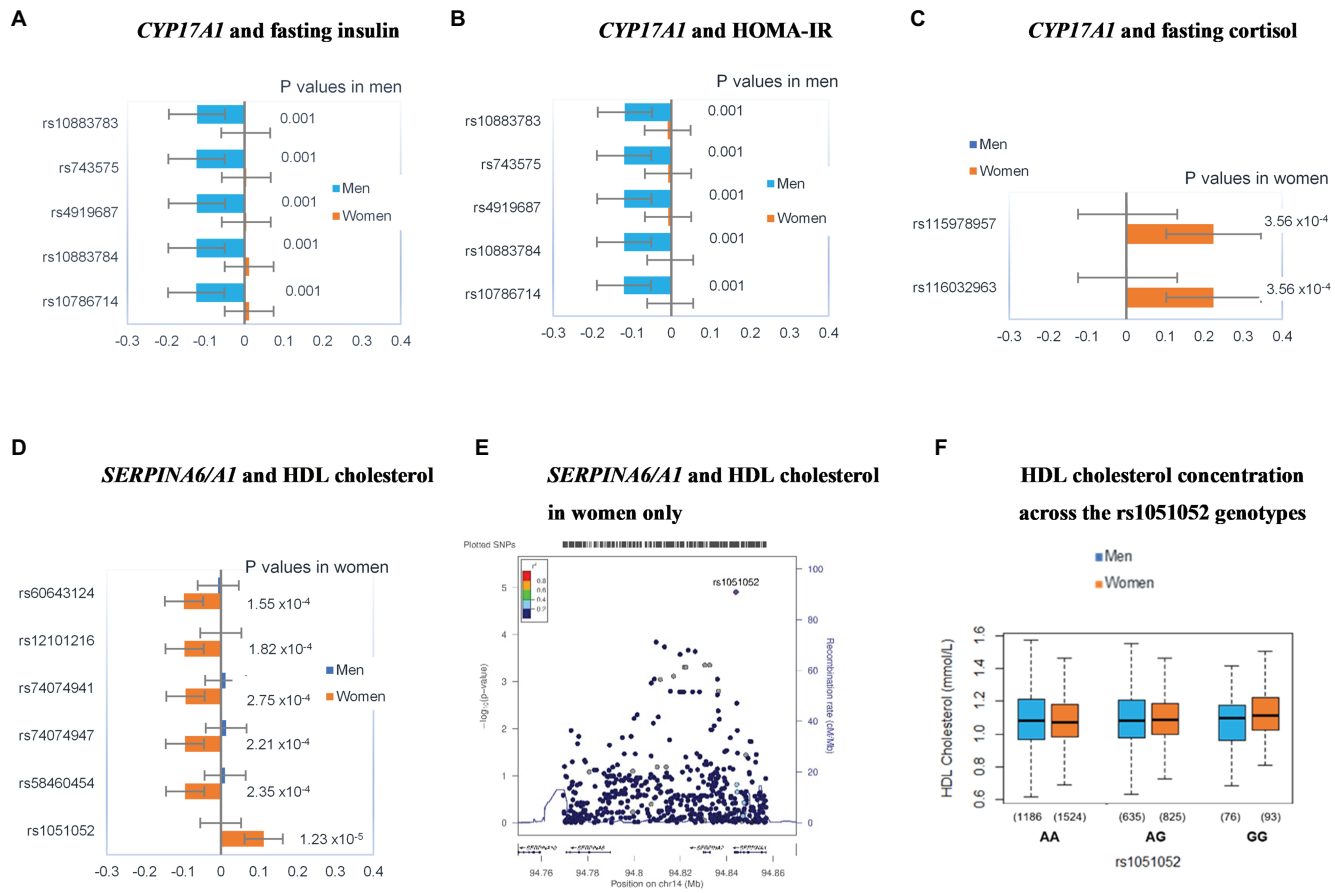
Sex-specific genetic associations between *CYP17A1* **(A-C)** and *SERPINA6/A1* **(D–F)** variants and components of the metabolic syndrome. Plots **(A-D)** show standardized beta coefficients for the linear regression models after adjusting for age, smoking, alcohol, and BMI. Error bars in these plots represent 95% CIs for the standardized beta coefficients. Values of *p* are for the respective beta coefficients. The LocusZoom plot summarizes the association between rs1051052 and higher HDL cholesterol concentrations in women **(E)**. The LocusZoom plot association data was plotted against the AFR LD backgrounds from the 1,000 Genomes Project phase 1 dataset. The rs1051052 SNP is represented by a purple diamond. Each SNP is colored according to the *r*^2^ value against rs1051052. SNPs with missing LD information are colored in gray. The box and whisker plot **(F)** shows the medians and IQRs of HDL cholesterol concentrations across the rs1051052 genotype groups in men and women. The number of observations in each group is shown in brackets.

In addition, there was evidence for sex interaction between five SNPs at *CYP17A1* and fasting insulin and HOMA-IR, as well as two *CYP17A1* SNPs and fasting cortisol. Likewise, there was evidence of a sex interaction for six SNPs at *SERPINA6/A1* and HDL cholesterol. Hence, the study sample was stratified by sex and the regression models that showed sex-interactions were repeated for men and women separately. These sex-stratified models are shown in Figure 3.

The associations between rs10883783-A, rs743575-G, rs4919687-A, rs10883784-T, and rs10786714-C at *CYP17A1*, and lower insulin and HOMA-IR were observed in men but not in women (Figures 3A,B). On the other hand, the associations between rs115978957-G and rs116032963-A at *CYP17A1*, and higher cortisol were observed in women but not in men (Figure 3C). Similarly, a set of SNPs, in *SERPINA6/A1*, rs60643124-G, rs12101216-T, rs74074941-T, rs74074947-T, and rs58460454-T showed association with lower HDL cholesterol in women only (Figure 3D). However, these signals did not remain significant after correction for multiple testing (all *p* > 2.28 × 10^−5^). Instead, the only association that remained significant even after adjusting for multiple testing was the women-specific association of rs1051052-G (*SERPINA6/A1* region) with higher HDL cholesterol (*p* = 1.23 × 10^−5^; Figure 3D). Figure 3E summarizes the LD architecture and association scores in the genomic region. The box and whisker plot (Figure 3F) shows the variation of HDL cholesterol between the three rs1051052 genotype groups.

### Frequencies of the Identified Effect Alleles

**Figure 4:**
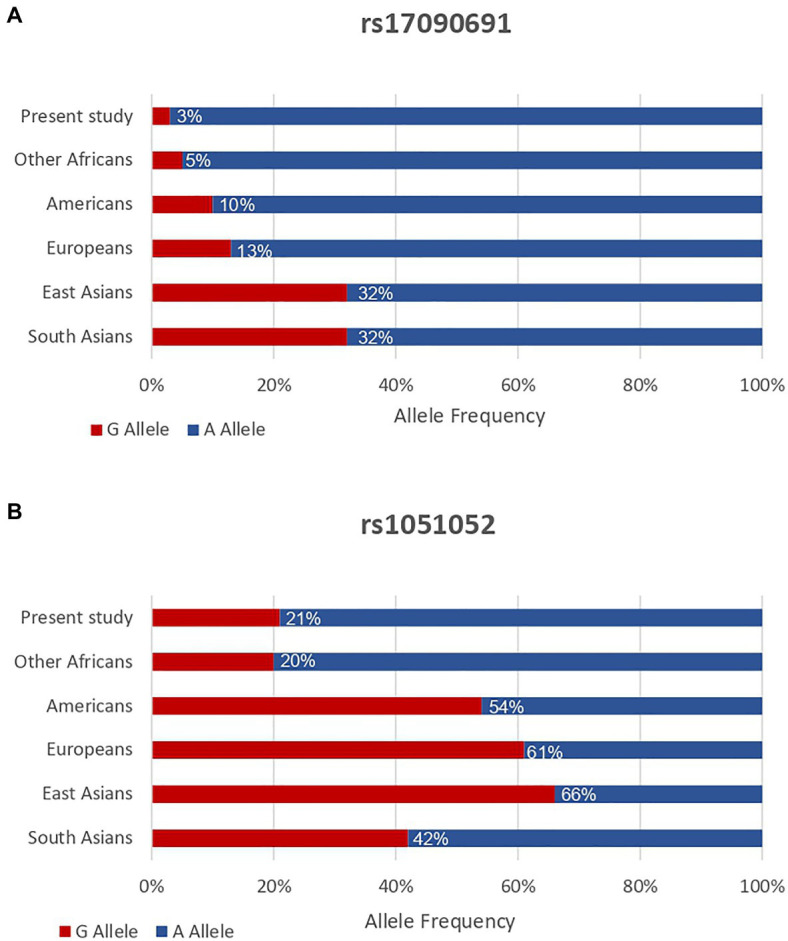
Allele frequencies for *SERPINA6/A1* rs17090691-G **(A)** and rs1051052-G **(B)** variants in the present study sample (*n* = 4,431) and other global populations. Minor allele frequencies from the other populations were obtained from the 1,000 Genomes Project phase 3 dataset. Other Africans include Yoruba in Ibadan (Nigeria), Luhya in Webuye (Kenya), Gambian in Western Divisions (Gambia), Mende (Sierra Leone), Esan (Nigeria), Americans of African Ancestry in SW (United States), and African Caribbeans (Barbados). Europeans include Utah Residents (CEPH) with Northern and Western European Ancestry, Toscani (Italy), Finnish (Finland), British (England and Scotland), and Iberian Population (Spain). Americans include Mexican Ancestry from Los Angeles (United States), Puerto Ricans (Puerto Rico), Colombians from Medellin (Colombia), and Peruvians from Lima (Peru). East Asians include Han Chinese in Beijing (China), Japanese in Tokyo (Japan), Southern Han Chinese, Chinese Dai in Xishuangbanna (China), and Kinh in Ho Chi Minh City (Vietnam). South Asians include Gujarati Indian from Houston (Texas), Punjabi from Lahore (Pakistan), Bengali (Bangladesh), Sri Lankan Tamil (the United Kingdom), and Indian Telugu (the United Kingdom).

In summary, only the relationship between rs17090691-G allele and higher diastolic blood pressure in the sample comprising both men and women, as well as between rs1051052-G allele and serum HDL cholesterol in women only, provided sufficient evidence of association after adjusting for multiple testing. The allele frequencies for these two SNPs (rs17090691 and rs1051052) from the *SERPINA6/A1* region were compared to those of other global populations (Americans, Europeans, East Asians, and South Asians) in Figure 4. The frequencies for the two identified effect alleles, rs17090691-G and rs1051052-G were similar to other African populations (3 vs. 5% and 21 vs. 20%, respectively), but lower than non-African populations (10–32 and 42–66%, respectively).

## Discussion

The present study reports two novel genetic associations between SNPs at *SERPINA6/A1* and key cardiometabolic risk factors in black South Africans. Specifically, the rs17090691-G allele was associated with higher diastolic blood pressure in a combined sample of men and women, while the rs1051052-G allele was associated with higher HDL cholesterol in women only. There were potential sex-specific associations between minor alleles of *CYP17A1* SNPs and lower insulin and HOMA-IR in men only, as well as between other *CYP17A1* SNPs and higher cortisol concentrations in women only. However, the evidence of these associations was not sufficient after adjusting for multiple testing. Further, there was no evidence to show that any of the identified genetic associations with diastolic blood pressure and HDL cholesterol were mediated by circulating glucocorticoids in black South Africans (Supplementary Table S2).

The associations between *CYP17A1* SNPs and measures of elevated blood pressure and adiposity have been previously reported in non-Africans (Levy et al., 2009; Hotta et al., 2012; Kelly et al., 2013; Franceschini et al., 2016), but *SERPINA6/A1* SNPs have not been previously associated with any cardiometabolic risk factor in any population. Instead, a GWAS in European men and women demonstrated that a SNP at *SERPINA6/A1*, represented by the rs12589136-T allele, was associated with higher circulating cortisol concentrations, while the rs2749527-T and rs11621961-T alleles at the same locus were associated with lower circulating cortisol concentrations (Boonen et al., 2013). Accordingly, a recent European study suggested that these previously identified *SERPINA6/A1* SNPs are likely to influence circulating cortisol concentrations by altering hepatic CBG expression (Crawford et al., 2021). Additionally, many cross-sectional studies have suggested that higher circulating cortisol concentrations are associated with an increased risk of developing the metabolic syndrome and its related cardiometabolic risk factors in both African and non-African populations (Walker et al., 2000; Reynolds et al., 2003; Ward et al., 2003). Based on these previous findings, the present study investigated whether common genetic variants at both *CYP17A1* and *SERPINA6/A1* loci are associated with the metabolic syndrome, its related cardiometabolic risk factors, and circulating glucocorticoids, in black South Africans.

To our knowledge, the rs17090691 SNP, which was associated with diastolic blood pressure in the present study, has not been associated with any other human trait. In contrast, the rs1051052-G allele, which was associated with higher HDL cholesterol in women in the present study, has not been associated with any cardiometabolic risk factors, but rather, has been associated with respiratory disorders. Specifically, the rs1051052-G allele was associated with higher odds of having childhood asthma in European girls and boys, but not their African counterparts (Baye et al., 2011), as well as chronic obstructive pulmonary disease in East Asian men and women (Zhong et al., 2018). Although, these traits are not directly related to the metabolic syndrome, similar mechanisms involving circulating glucocorticoid concentrations may also be involved (Baye et al., 2011; Zhong et al., 2018).

Circulating glucocorticoids are known to increase blood pressure *via* several mechanisms, including impairment of nitric oxide-mediated renal vasodilation in the kidneys (De Matteo and May, 1997). Accordingly, higher concentrations of circulating cortisol, the primary glucocorticoid in humans, have been consistently shown to be associated with measures of elevated blood pressure in both men and women, regardless of ethnicity (Filipovský et al., 1996; Phillips et al., 1998; Walker et al., 2000; Reynolds et al., 2003; Ward et al., 2003; Weigensberg et al., 2008; Adam et al., 2010; Park et al., 2011; Tolmay et al., 2012; Guzzetti et al., 2014; Constantinopoulos et al., 2015; Schutte et al., 2016). As the rs17090691 SNP is intergenic between *SERPINA6* and *SERPINA1*, it is likely that this SNP is in LD with the causal variant. While common variants generally contribute a modest amount to complex phenotypes such as diastolic blood pressure, functional studies should still investigate whether having the identified effect allele is associated with expression of CBG and/or alpha-1 antitrypsin, to assess the mechanisms involved. Notably, the identified rs17090691-G allele (effect allele) has a higher frequency in populations of non-African ancestry (10–32 vs. 3–5%). Thus, a replication study that includes non-African populations is required to understand whether the association occurs in other ethnic groups.

The two *SERPINA6/A1* SNPs associated with cardiometabolic outcomes in the present study localize to a separate haplotype block from those previously associated with cortisol in Europeans (Bolton et al., 2014), and there are currently no reported functional studies for these SNPs. Furthermore, we did not find an association between the identified SNPs and circulating glucocorticoid concentrations in the sub-sample of men and women from Soweto. Notably, circulating glucocorticoid concentrations change dynamically and are influenced by several known and unknown confounders (Kelly et al., 2008). For example, circulating glucocorticoid concentrations exhibit a diurnal nature, such that peaks are seen 30–45 min after awakening and the concentrations decline throughout the day (Clow et al., 2010). Morning glucocorticoid concentrations were measured in this study, but not all samples were taken at the same time. Although, blood sampling time was corrected for this may still have influenced the findings. Further, it is also possible that the identified *SERPINA6/A1* SNPs were associated with the variability in daily circulating glucocorticoid concentrations instead of the morning concentrations that were measured in the present study. Furthermore, our available sample size for glucocorticoid determination was relatively small and the lack of associations with glucocorticoids may have resulted from low statistical power. For these reasons, a larger and more detailed study in Africans is required to explore whether circulating glucocorticoids mediated the observed genetic associations.

The rs1051052-G allele, which was associated with higher HDL cholesterol in women in the present study, resides within the 3' untranslated region of *SERPINA1*. However, no functional studies have been undertaken to assess its influence on the genetic expression and function of its protein product, alpha-1 antitrypsin. Hence, the molecular consequences of rs1051052-G allele also remain to be explored. Nevertheless, this is the first study to show that this minor allele is associated with higher HDL cholesterol in women of African ancestry. These findings support the hypothesis that the often-observed sex differences in lipid and lipoprotein metabolism may be partly related to glucocorticoid signaling, which is often not considered as a potential confounder. However, the frequency of this allele is much lower in populations of African ancestry than in non-Africans (20–21 vs. 42–66%). Notably, black South African women typically present with lower HDL cholesterol compared to their European ancestry counterparts (Després et al., 2000; Punyadeera et al., 2001; Sumner and Cowie, 2008; Goedecke et al., 2010; Sliwa et al., 2012). However, results from the present study support previous findings that HDL cholesterol concentrations are similar between African men and women (Kodaman et al., 2016; van der Linden et al., 2019), an observation which is different to that reported in populations of European Ancestry (Balder et al., 2017). While sex and ethnic differences in serum lipid profiles are thought to be driven by differences in central adiposity, in particular differences in visceral adipose tissue (VAT; Keswell et al., 2016), lifestyle factors may also influence the differences in lipid profiles (Goedecke et al., 2010; Keswell et al., 2016; van der Linden et al., 2019). Indeed, HDL cholesterol concentrations are associated with alcohol intake, physical activity, and importantly, inflammation (Choi and Seeger, 2005; Keswell et al., 2016). Hence, exploring gene-environment interactions in African populations is required to understand sex and ethnic differences in serum lipid profiles.

Although, we detected some evidence for the associations between some of the *CYP17A1* SNPs and lower insulin and HOMA-IR in men only, and some of the *CYP17A1* SNPs with cortisol concentrations in women only, these signals were not conclusive as they did not remain significant after stringent Bonferroni corrections. Moreover, these sex-specific associations were possibly confounded by the vast differences in body fat between men and women in this cohort (obesity prevalence = 53.4% in women vs. 13.7% in men). As these sex differences are characteristic of the black South African population (Pillay et al., 2015), a larger sample size would be required to confirm the sex-specific associations between *CYP17A1* SNPs and insulin and HOMA-IR, as well as glucocorticoid concentrations in African men and women, respectively.

The lack of evidence of association between previously identified *CYP17A1* and *SERPINA6/A1* SNPs, and the tested cardiometabolic risk factors in the present study, was likely attributed to lower minor allele frequencies (Supplementary Table S3) in combination with the distinct metabolic profile of the studied population (e.g., almost half of the participants had the metabolic syndrome), compared to non-Africans. Due to these factors, a larger sample size may be required to replicate the previously identified associations. Nevertheless, investigating a black South African population of middle-aged men and women is a major strength of the present study. Considering the possibility that the observed associations may be African-specific, these novel genetic associations may have been previously missed, since most genetic association studies have been conducted in Europeans. A recent review has shown that the composition of participants in previously published GWAS is largely Eurocentric, with approximately 78% European, 10% Asian, and only 2% African (Sirugo et al., 2019). Therefore, large scale studies such as ours are necessary to test the transferability of European cohort-based signals to other ethnic groups and geographic regions. Moreover, the previous genetic association studies in Africans were limited by the use of genotyping arrays that are not designed to capture the diversity observed in the African genome (Pillay et al., 2015; Hendry et al., 2018). The use of an African-centric array and African enriched imputation panel enabled us to capture genetic diversity around these two loci more efficiently. The relatively larger sample size allowed for the inclusion of sufficient men and women participants in the present study for sex specific association testing. Notably, several explanations, including multifactorial models (interactions between biological and environmental factors; Reich et al., 1975), the sex-dependent liability threshold (Carter and Evans, 1969), and sex-disparities in the underlying genetic architecture (Khramtsova et al., 2019), have been proposed to explain sex-specific genetic associations in human phenotypes.

The present study also has some limitations. The statistical power of the study was limited by a moderate sample size at the beginning of the study and the criteria used to select the final participants. Hence, lack of genetic associations with some of the key cardiometabolic risk factors, and with the odds of having the metabolic syndrome, may be attributed to a lack of statistical power. Moreover, we did not adjust for possible population substructure within the black South African population, which could have influenced some of the genetic association results (Sengupta et al., 2020). Furthermore, circulating glucocorticoid concentrations were only measured in a subset of the study population and the influence of the circadian rhythm could not be assessed. Future studies should also measure the diurnal curves to draw better conclusions regarding mediation by circulating glucocorticoids.

## Conclusion

The present study reports novel genetic associations between two SNPs at *SERPINA6/A1* and key cardiometabolic risk factors in black South African men and women. These findings support the hypothesis that common variants in the *SERPINA6/A1* locus are associated with key cardiometabolic risk factors in humans. Future functional studies are also required to confirm the role of the identified SNPs in the metabolic syndrome and assess whether these associations are mediated by circulating glucocorticoids.

## Data Availability Statement

The original contributions presented in the study are included in the article/Supplementary Material, further inquiries can be directed to the corresponding authors.

## Ethics Statement

The studies involving human participants were reviewed and approved by University of Witwatersrand Human Research Ethics Committee (Medical). The patients/participants provided their written informed consent to participate in this study.

## Author Contributions

SD, JG, ZL, AC, AAC, MR, and BW were responsible for the conception and planning of the study. MR, LM, JG, NC, and SN were involved in sample and data collection. SD was responsible for measuring serum glucocorticoid concentrations. SD, AC, JG, and ZL were involved in the data analyses. SD, AC, MR, LM, SN, NC, AAC, BW, ZL, and JG were involved in the interpretation of the results and the writing of the manuscript. All authors contributed to the article and approved the submitted version.

## Conflict of Interest

The authors declare that the research was conducted in the absence of any commercial or financial relationships that could be construed as a potential conflict of interest.

## Publisher’s Note

All claims expressed in this article are solely those of the authors and do not necessarily represent those of their affiliated organizations, or those of the publisher, the editors and the reviewers. Any product that may be evaluated in this article, or claim that may be made by its manufacturer, is not guaranteed or endorsed by the publisher.

## Footnotes

^1^ https://www.dtu.ox.ac.uk/homacalculator
