# Supplementary material for: Associations Between CYP17A1 and SERPINA6/A1 Polymorphisms, and Cardiometabolic Risk Factors in Black South Africans
Source: Front Genet. 2021 Aug 13;12:687335. doi: 10.3389/fgene.2021.687335 (PMC8414563; doi:10.3389/fgene.2021.687335)
Supplement: Supplementary file 1 [file Table_1.DOCX]

|  | **Combined sexes** | | | **Men only** | | | **Women only** | | |
| --- | --- | --- | --- | --- | --- | --- | --- | --- | --- |
|  | **Black SA AWI-Gen (n =4431)** | **Glucocorticoid Sub-Sample (n =877)** | **P value** | **Black SA AWI-Gen (n =1918)** | **Glucocorticoid Sub-Sample (n =304)** | **P value** | **Black SA AWI-Gen (n =2513)** | **Glucocorticoid Sub-Sample (n =573)** | **P value** |
| **Age (years)** | 52 (46-58) | 52 (47-57) | 0.144 | 52 (46-58) | 52 (47-58) | 0.094 | 52 (46-58) | 52 (47-56) | 0.485 |
| **BMI (kg/m^2^)** | 27.2 (22.2-32.7) | 30.3 (24.6-35.4) | <0.001 | 23.2 (20.1-27.4) | 25.2 (21.0-30.0) | <0.001 | 30.5 (25.8-35.5) | 32.9 (28.6-37.7) | <0.001 |
| **Waist (cm)** | 95.8 (89.4-101.5) | 95.5 (86.5-105.0) | 0.930 | 88.0 (78.0-89.8) | 93.0 (81.5-103.8) | 0.914 | 98.0 (92.3-103.2) | 96.5 (88.5-105.4) | 0.115 |
| **Smoking (n/N (%))** | 597 / 3108 (19.2) | 174 / 771 (22.6) | 0.041 | 538 / 1078 (49.9) | 141 / 276 (51.1) | 0.778 | 59 / 2030 (2.9) | 33 / 495 (6.7) | 0.001 |
| **Alcohol (n/N (%))** | 1292 / 2502 (51.6) | 288 / 569 (50.6) | 0.693 | 954 / 1168 (81.7) | 198 / 276 (71.7) | 0.003 | 338 / 1334 (25.3) | 90 / 293 (30.7) | 0.069 |
| Continuous data presented as **median (IQR: Interquartile range)** and categorical data presented as number of prevalent / total number of observations **(n/N)** and percentage **(%)**. **Wilcoxon Rank Sum** and C**hi-square test** were used to statistically compare the continuous and categorical variables, respectively, between the black South African AWI-Gen sample and the glucocorticoid sub-sample. **SA:** South African; **p value**: p value for the statistical difference between the sample groups; **BMI:** Body Mass Index; **Waist:** Waist circumference. Smoking and alcohol statuses were self-reported during interview sessions. | | | | | | | | | |

**Table S1: A comparison between the black South African AWI-Gen sample and the glucocorticoid subsample.**
