# Supplementary material for: Associations Between CYP17A1 and SERPINA6/A1 Polymorphisms, and Cardiometabolic Risk Factors in Black South Africans
Source: Front Genet. 2021 Aug 13;12:687335. doi: 10.3389/fgene.2021.687335 (PMC8414563; doi:10.3389/fgene.2021.687335)
Supplement: Supplementary file 2 [file Table_2.docx]

**Table S2: Lack of evidence of previously observed associations in Black South Africans.**

| **Locus** | | **Previous/present associations** | **Lack of association in this study** | | | |
| --- | --- | --- | --- | --- | --- | --- |
|  |  |  | **Phenotype** | **Beta/OR** | **95%CI** | **p** |
| ***CYP17A1*** | rs1004467 | Measures of blood pressure in Europeans and East Asians | Diastolic blood pressure | 0.003 | -0.010, 0.017 | 0.617 |
|  |  |  | Systolic blood pressure | 0.000 | 0.000, 0.000 | 0.258 |
|  | rs2486758 | Circulating corticosterone concentrations and the corticosterone/cortisol ratio in Europeans | Corticosterone | 0.079 | -0.020, 0.177 | 0.119 |
|  |  |  | Cortisol | 0.122 | 0.028, 0.215 | **0.011** |
|  |  |  | Corticosterone/cortisol | -0.087 | -0.191, 0.017 | 0.102 |
| ***SERPINA6/A1*** | rs17090691 | Diastolic blood pressure in men and women of the present study | Metabolic Syndrome | 0.973 | 0.708, 1.337 | 0.865 |
|  |  |  | Corticosterone | 0.069 | -0.044, 0.181 | 0.231 |
|  |  |  | Cortisol | 0.015 | -0.089, 0.118 | 0.781 |
|  |  |  | Corticosterone/cortisol | 0.036 | -0.083, 0.155 | 0.554 |
|  | rs1051052 | HDL cholesterol concentrations in women of the present study | Metabolic Syndrome | 0.917 | 0.807, 1.042 | 0.184 |
|  |  |  | Corticosterone | -0.014 | -0.070, 0.041 | 0.610 |
|  |  |  | Cortisol | -0.030 | -0.083, 0.023 | 0.272 |
|  |  |  | Corticosterone/cortisol | 0.017 | -0.043, 0.077 | 0.577 |
|  | rs11621961 | Circulating cortisol concentrations in Europeans | Corticosterone | 0.001 | -0.005, 0.006 | 0.761 |
|  |  |  | Cortisol | -0.043 | -0.110, 0.023 | 0.204 |
|  |  |  | Corticosterone/cortisol | -0.016 | -0.093, 0.060 | 0.676 |
|  | rs12589136 | Circulating cortisol concentrations in Europeans | Corticosterone | 0.001 | -0.005, 0.006 | 0.762 |
|  |  |  | Cortisol | 0.017 | -0.039, 0.074 | 0.549 |
|  |  |  | Corticosterone/cortisol | -0.014 | -0.078, 0.050 | 0.662 |
|  | rs2749529 | Circulating cortisol concentrations in Europeans | Corticosterone | 0.049 | -0.024, 0.123 | 0.188 |
|  |  |  | Cortisol | 0.014 | -0.054, 0.082 | 0.685 |
|  |  |  | Corticosterone/cortisol | -0.007 | -0.087, 0.073 | 0.862 |
|  | rs2749527 | Circulating cortisol concentrations in Europeans | Corticosterone | 0.061 | -0.057, 0.180 | 0.310 |
|  |  |  | Cortisol | 0.021 | -0.100, 0.142 | 0.738 |
|  |  |  | Corticosterone/cortisol | 0.003 | -0.136, 0.142 | 0.967 |

**Beta:** Unstandardized beta coefficient for the linear regression model; **95% CI:** 95% confident intervals
