# Supplementary material for: Associations Between CYP17A1 and SERPINA6/A1 Polymorphisms, and Cardiometabolic Risk Factors in Black South Africans
Source: Front Genet. 2021 Aug 13;12:687335. doi: 10.3389/fgene.2021.687335 (PMC8414563; doi:10.3389/fgene.2021.687335)
Supplement: Supplementary file 3 [file Table_3.DOCX]

**Table S3: Minor allele frequency comparison of the previously identified SNPs to black South Africans.**

| SNP | Minor allele | Minor allele frequency | | | | | |
| --- | --- | --- | --- | --- | --- | --- | --- |
|  |  | Present study | Other Africans | Europeans | Americans | East Asians | South Asians |
| rs1004467 | G | 0.13 | 0.19 | 0.09 | 0.21 | 0.35 | 0.26 |
| rs2486758 | C | 0.04 | 0.04 | 0.23 | 0.24 | 0.19 | 0.25 |
| rs11621961 | T | 0.13 | 0.16 | 0.36 | 0.45 | 0.21 | 0.25 |
| rs12589136 | T | 0.17 | 0.15 | 0.2 | 0.13 | 0.4 | 0.38 |
| rs2749529 | A | 0.08 | 0.13 | 0.56 | 0.71 | 0.64 | 0.31 |
| rs2749527 | T | 0.02 | 0.10 | 0.51 | 0.68 | 0.64 | 0.31 |

| **SNP:** Single Nucleotide Polymorphism. Minor allele frequencies from the other populations were obtained from the **1000 genomes phase 3** dataset. **Other Africans** include Yoruba in Ibadan (Nigeria), Luhya in Webuye (Kenya), Gambian in Western Divisions (Gambia), Mende (Sierra Leone), Esan (Nigeria), Americans of African Ancestry in SW (USA) and African Caribbeans (Barbados). **Europeans** include Utah Residents (CEPH) with Northern and Western European Ancestry, Toscani (Italia), Finnish (Finland), British (England and Scotland) and Iberian Population (Spain). **Americans** include Mexican Ancestry from Los Angeles (USA), Puerto Ricans (Puerto Rico), Colombians from Medellin (Colombia) and Peruvians from Lima (Peru). **East Asians** include Han Chinese in Beijing (China), Japanese in Tokyo (Japan), Southern Han Chinese, Chinese Dai in Xishuangbanna (China) and Kinh in Ho Chi Minh City (Vietnam). **South Asians** include Gujarati Indian from Houston (Texas), Punjabi from Lahore (Pakistan), Bengali (Bangladesh), Sri Lankan Tamil (the United Kingdom) and Indian Telugu (the United Kingdom). |
| --- |
